# 3D Modeling and Printing in Congenital Heart Surgery: Entering the Stage of Maturation

**DOI:** 10.3389/fped.2021.621672

**Published:** 2021-02-05

**Authors:** Shi Joon Yoo, Nabil Hussein, Brandon Peel, John Coles, Glen S. van Arsdell, Osami Honjo, Christoph Haller, Christopher Z. Lam, Mike Seed, David Barron

**Affiliations:** ^1^Department of Diagnostic Imaging, The University of Toronto, Toronto, ON, Canada; ^2^Department of Paediatrics–Division of Cardiology, The University of Toronto, Toronto, ON, Canada; ^3^Center for Image Guided Innovation and Therapeutic Intervention, The University of Toronto, Toronto, ON, Canada; ^4^Department of Surgery-Division of Cardiovascular Surgery, Hospital for Sick Children, The University of Toronto, Toronto, ON, Canada; ^5^Department of Surgery, David Geffen School of Medicine at the University of California Los Angeles, Los Angeles, CA, United States; ^6^Department of Surgery, Mattel Children's Hospital at UCLA, Los Angeles, CA, United States

**Keywords:** congenital heart surgery, 3D modeling, 3D printing, hands-on surgical training, surgical simulation, education

## Abstract

3D printing allows the most realistic perception of the surgical anatomy of congenital heart diseases without the requirement of physical devices such as a computer screen or virtual headset. It is useful for surgical decision making and simulation, hands-on surgical training (HOST) and cardiovascular morphology teaching. 3D-printed models allow easy understanding of surgical morphology and preoperative surgical simulation. The most common indications for its clinical use include complex forms of double outlet right ventricle and transposition of the great arteries, anomalous systemic and pulmonary venous connections, and heterotaxy. Its utility in congenital heart surgery is indisputable, although it is hard to “scientifically” prove the impact of its use in surgery because of many confounding factors that contribute to the surgical outcome. 3D-printed models are valuable resources for morphology teaching. Educational models can be produced for almost all different variations of congenital heart diseases, and replicated in any number. HOST using 3D-printed models enables efficient education of surgeons in-training. Implementation of the HOST courses in congenital heart surgical training programs is not an option but an absolute necessity. In conclusion, 3D printing is entering the stage of maturation in its use for congenital heart surgery. It is now time for imagers and surgeons to find how to effectively utilize 3D printing and how to improve the quality of the products for improved patient outcomes and impact of education and training.

## Introduction

Congenital heart diseases (CHDs) are structural heart diseases that are characterized by extremely wide anatomical variations and complexity with a number of individual pathologic entities that are only rarely encountered in practice. CHDs usually require surgical treatment or catheter-based intervention that requires proper understanding of three-dimensional (3D) surgical anatomy. Understanding of these complex structures requires both 2D and 3D imaging. While 2D imaging is for exploration of the composition of a 3D structure, 3D imaging is for intuitive understanding of a structure's shape. The more complex the structure, the higher the need for 3D demonstration. This can be represented on a computer screen, in a simulated environment (i.e., virtual/augmented or mixed reality) or through physical 3D-printed models. Among these three paradigms of 3D demonstration, 3D printing allows the most realistic perception of the pathology without the requirement of devices such as a computer screen or virtual headset. The tactile properties of 3D-printed models enhance understanding of the anatomy with clarity and unambiguity, minimizing the risks of misinterpretation and miscommunication. In addition, the models provide an opportunity for hands-on simulation of surgical or interventional procedures, which is not available on the other platforms.

In the last two decades, 3D printing has become practically applicable and valuable for congenital heart surgery (1–9). In this review article, we focus our discussion on current applications, limitations and the future direction of 3D printing in congenital heart surgery. The techniques for 3D modeling and printing are well-presented elsewhere (1, 10, 11).

## Applicable Imaging Modalities

Contrast-enhanced computed tomography (CT) and magnetic resonance (MR) angiograms provide the 3D image data that are best suited for 3D modeling and printing owing to excellent contrast and spatial resolutions. For the best quality of images and 3D printing, imaging should be performed with electrocardiographic (ECG) gating and breath-holding (for CT) or respiration navigation (for MR). As cardiac surgery is usually performed with cardioplegia, ECG-gating is preferred to target end diastole. When ECG-gating or breath-holding/respiration navigation is not applicable, non-ECG-gated imaging during quiet respiration is acceptable with the detail compromised. CT provides a high spatial resolution ranging between 0.5 and 0.65 mm, while the spatial resolution of MR is limited to 1–2 mm. In both CT and MR, homogeneous opacification of the cardiac structures is important as postprocessing for 3D printing relies primarily on thresholding based on signal intensity. Although MR requires a long imaging time, homogeneity of contrast enhancement is easier to achieve at MR than at CT. CT images reconstructed from rotational angiograms obtained during cardiac catheterization are also applicable for 3D printing (12). 3D echocardiography is an excellent tool for 3D visualization of cardiovascular structures on a computer screen (13). However, ultrasound imaging is inherently associated with strong artifacts from air and bone, which hampers utilization of echocardiograms for 3D printing. Furthermore, the field of view at echocardiography is limited to the availability of the window for sound propagation. Therefore, echocardiogram-based 3D printing has been limited mostly to demonstration of cardiac valves and guidance for device closures (13–15). The strengths of CT or MR and echocardiograms can be integrated for so-called hybrid 3D printing (16).

Acquired DICOM (Digital Imaging and COmmunication in Medicine) data are processed for 3D modeling using threshold-based segmentation and additional manual editing (1–4). The segmented DICOM data are then converted to the STL (Stereolithography or Standard Tessellation Language) or other file format for 3D modeling and printing.

## Current Applications

Applications of 3D printing in congenital heart surgery can be divided into the clinical applications for surgical decision making and planning, preoperative surgical simulation and computer-aided design of the required patches and baffles, and the applications for education and surgical skill training (Table 1).

**Table 1:** Applications of 3D printing in congenital heart surgery.

Clinical • Surgical decision making and planning • Surgical simulation • Computer-aided design of surgical baffles and patches
Educational • Hands-on surgical training (HOST) • Congenital heart morphology teaching • Patient and family education

### Surgical Decision Making and Planning

**Figure 1:**
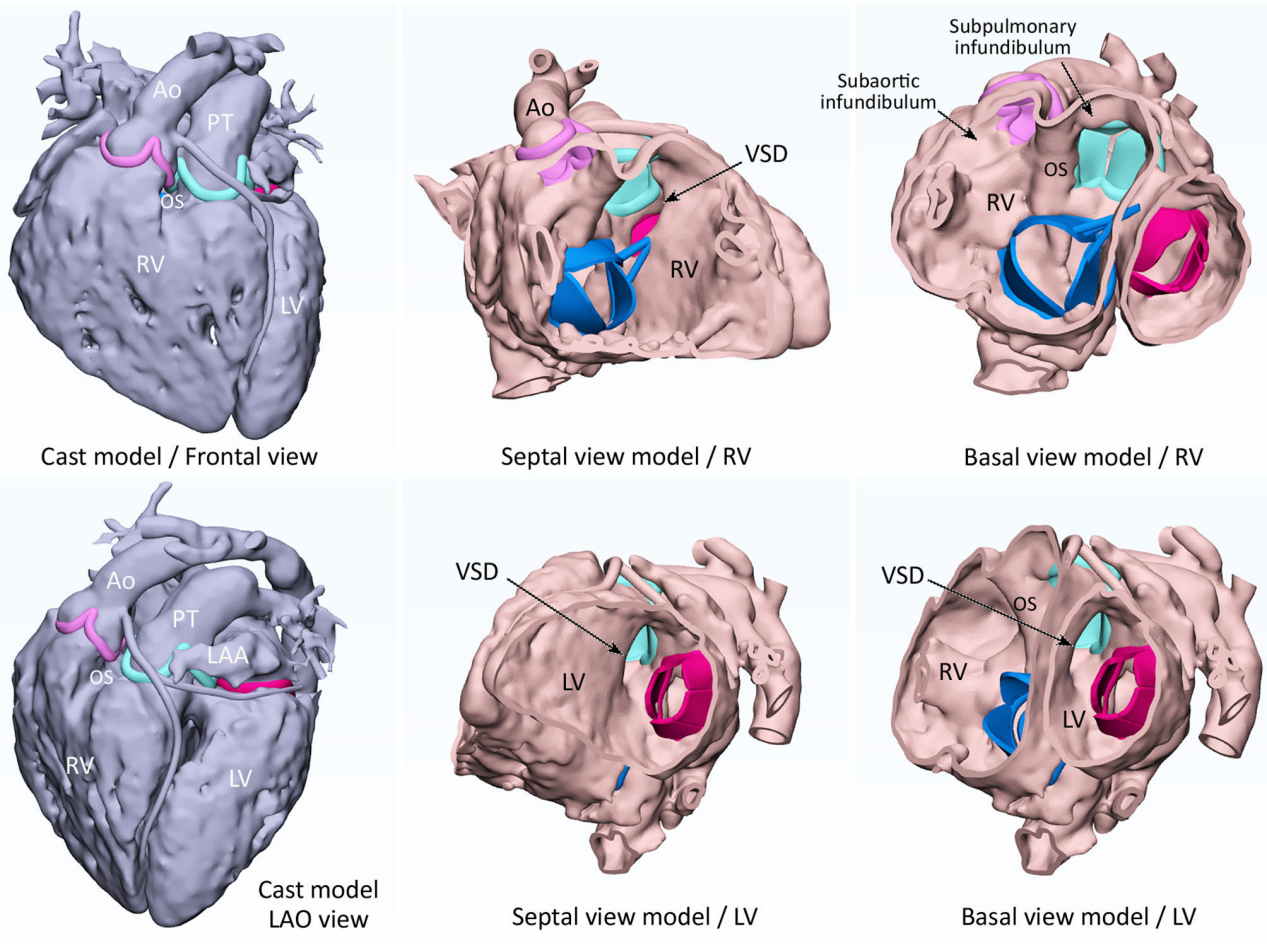
3D modeling of cast (left-hand panels) and endocardial surface anatomy (middle and right-hand panels) for double outlet right ventricle with a subpulmonary ventricular septal defect (VSD). A cast is modeled from CT images by signal intensity-based thresholding for contrast-enhanced blood pool. By hollowing the cast model toward outside, a negative of the cast is produced to replicate the endocardial surface anatomy. The endocardial surface represents true anatomy, while the outer surface does not. The sites of attachments of the cardiac valve leaflets are marked as ridges and the graphically designed leaflets and chords are added for reference. Ao, aorta; LAA, left atrial appendage; LAO, left anterior oblique; LV, left ventricle; OS, outlet septum; PT, pulmonary trunk; RV, right ventricle; SVC, superior vena cava.

A choice between biventricular and univentricular repairs in complex CHDs is frequently a binary decision that determines the ultimate fate of the patient's health (17, 18). The decision requires precise understanding of: (1) the feasibility of separation of the systemic and venous flows within the limited space of cardiac chambers, (2) establishment of patent ventricular outflow tracts, ideally, with competent valves, and (3) construction of unobstructed pulmonary arteries and aorta/aortic arch. While the decision could be reached with 2D images alone, 3D imaging facilitates understanding of the surgical anatomy in complex cases (7–9, 18). While 3D modeling of the blood pool provides an excellent overview of the pathology, 3D endocardial surface imaging reproduces the surgical scenes of the opened cardiac cavities (Figure 1) (1, 11).

**Figure 2:**
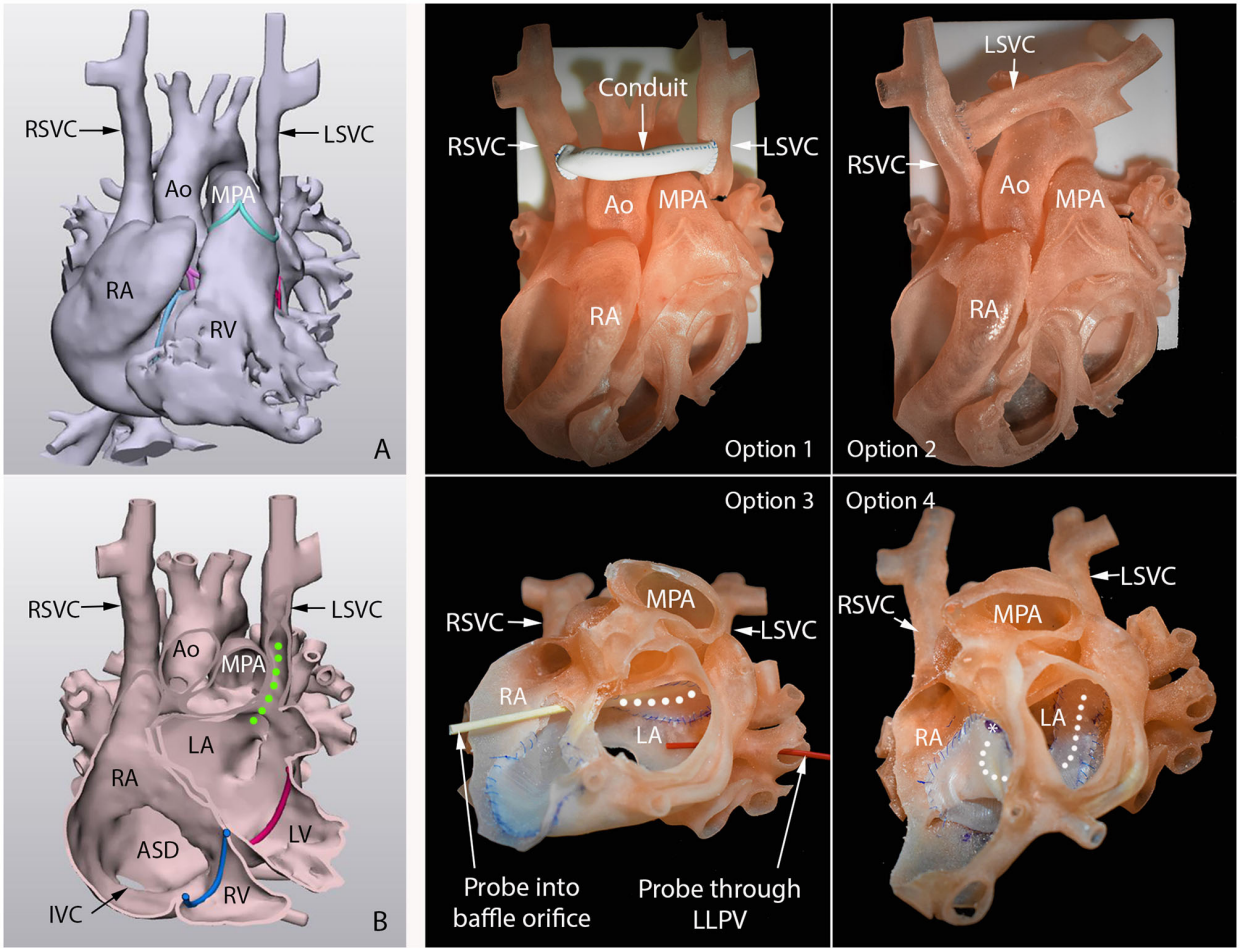
3D-printed surgical simulation model for a patient with so-called Raghib syndrome (unroofed coronary sinus with persistent left superior vena cava). Left hand panels are frontal views of the 3D-reconstructed cast image **(A)** and endocardial surface image **(B)**. Green dotted line shows the path of the left superior vena cava (LSVC) that connects to the roof of the left atrium (LA). Middle and right-hand panels show four surgical options practiced on the 3D-printed models. Option 1, graft interposition between right (RSVC) and LSVC. Option 2, direct anastomosis of disconnected LSVC to RSVC without graft. Option 3, intraatrial baffling of LSVC orifice to right atrium (RA) along the roof of the left atrium (LA). Option 4, intraatrial baffling of LSVC orifice to RA along the floor of the LA. The course of the baffle is marked with white dotted line. Option 3 was chosen for surgical treatment (29).

Double outlet right ventricle (DORV) is by far the most common referral indication for 3D printing (11, 19–23). DORV is not a single pathologic entity but a collective term for various malformations with a unifying feature of origin of more than half of both arterial trunks from the right ventricle (24). Although there are more common forms of DORV such as those with a ventricular septal defect (VSD) in strictly subaortic or subpulmonary location, a number of cases show non-classic features. Naming a VSD as remote in DORV may discourage surgeons to proceed with a biventricular repair. However, biventricular repair can be an achievable goal in a significant number of DORVs with a remote VSD (25). 3D-printed models are very helpful for optimal pre-operative decision making in these cases. Similarly, complex forms of transposition of the great arteries are often referred for 3D printing to assess the feasibility of options such as intraventricular baffling of the VSD to an arterial valve, a Nikaido procedure, or double-root translocation (26–28). Complex systemic and/or pulmonary venous anatomy is another frequent referral indication. Examples include persistent left superior vena cava with unroofed coronary sinus (so-called Raghib syndrome), sinus venosus type atrial septal defect, and complex venous anatomy in heterotaxy in which intraatrial or extracardiac baffling of an anomalous vein is required (Figure 2) (29–33). Usefulness of 3D printing is not limited to the examples listed above but is applicable in any situation where the decision cannot be confidently made with 2D imaging findings alone.

### Surgical Simulation

Surgical or interventional procedures can be simulated on 3D-printed models made of soft materials (Figure 2) (11, 29–31). Commercially available soft materials such as TangoPlus, Agilus and TissueMatrix (Stratasys, Edina, Minnesota, US) are more pliable than human myocardial and endocardial tissues. Most surgeons find that suturing is more difficult on models than on human tissue. However, incision and suturing can be simulated on the models with caution. The surgeons have the opportunity to practice the intended surgical procedure on the models printed from the patient's own image data if they are not experienced in or familiar with the procedure. 3D-printed models can be used for simulation of minimally invasive surgery in which the field and angle of view is limited (34). Preoperative simulation for trainee surgeons is of paramount importance in order to minimalize any potential risk to patient safety and to increase their confidence and technical skills associated with the procedure.

For experienced surgeons, simulation can be beneficial in cases where there are more than one potential surgical approach to treating the defect. Usually these complex decisions are made based on the surgeon's own experience and training background as well as by referencing the literature. For complex cases where the surgical treatment strategy can be controversial, the surgeon may use 3D models to simulate different surgical strategies to help refine what is the best applicable procedure for the given patient's anatomy (Figure 2). Furthermore, 3D-printed models can be used for the development and trial of newly invented procedures. The predicted anatomical and hemodynamic results of the simulated surgery can be replicated by 3D modeling/printing and 4D flow MR imaging or computational fluid dynamics in order to objectively assess surgical outcome (35).

### Computer-Aided Design of Surgical Baffles and Patches

Traditionally, patches for septal defect closure, intraventicular baffles and vascular augmentation patches are trimmed during surgical procedures. While simple closure patches are easy to trim, complex patches and baffles require surgeon's best guess of the result for appropriate trimming and application. Such empirical approaches to complex surgical procedures are hard to be standardized and frequently associated with unsatisfactory anatomical results (36, 37). The required baffles and patches can be graphically designed for the patient's specific anatomy to predict the surgical results such as patency of the pathway without obstruction or unfavorably dilated lumen, adequacy of the volume of the remaining part of the ventricle after application of a large intraventricular baffle, and spatial relationship between the baffles/patches and the cardiac valves (11, 38, 39). A sterilizable template can be graphically designed and 3D printed to guide trimming of a baffle or patch at surgery.

### Hands-On Surgical Training (HOST)

Surgical training for CHDs is challenging and requires a long training period because of relative rarity of individual lesions and demanding surgical techniques. Furthermore, intraoperative training presents a potential risk to patient safety. As a consequence, surgical trainees have been provided with limited operative experience in congenital heart surgery. Therefore, surgical simulation has long been sought to develop surgeons' technical skills without compromising patient safety (40, 41). Until recently, available resources for surgical simulation have been limited to the hearts and vessels of living or sacrificed animals, pathologic heart specimens and plastic replicas (42). As these resources rarely represent the pathological anatomy of CHDs, surgical simulation has been applicable for the procedures that can be simulated on a normal heart, such as arterial switch, arterial root translocation and the Ross procedure (43). 3D printing with soft materials has brought a new opportunity for the training of various surgical procedures. With improved imaging technology and widespread utilization of CT and MR, image data for 3D modeling and printing are readily available for most CHDs. Furthermore, 3D printing allows reproduction of any number of models for unlimited availability. Since its introduction in 2015, Hands-On Surgical Training (HOST) courses using 3D-printed models have increasingly been popular among surgeons and trainees in congenital heart surgery (44–46). Most participants of the HOST courses appreciated the educational value of the 3D-printed models in improving their surgical skills. Most of them also strongly agreed that the HOST courses should be included in the curriculum of CHD surgical training. The authors' institution holds monthly HOST sessions for its own trainees with excellent feedback from both trainees and proctors (47). This platform also allows surgeons to practice the procedures with proctor's supervision via an online format or to practice independently with pre-recorded training videos to reference from.

**Figure 3:**
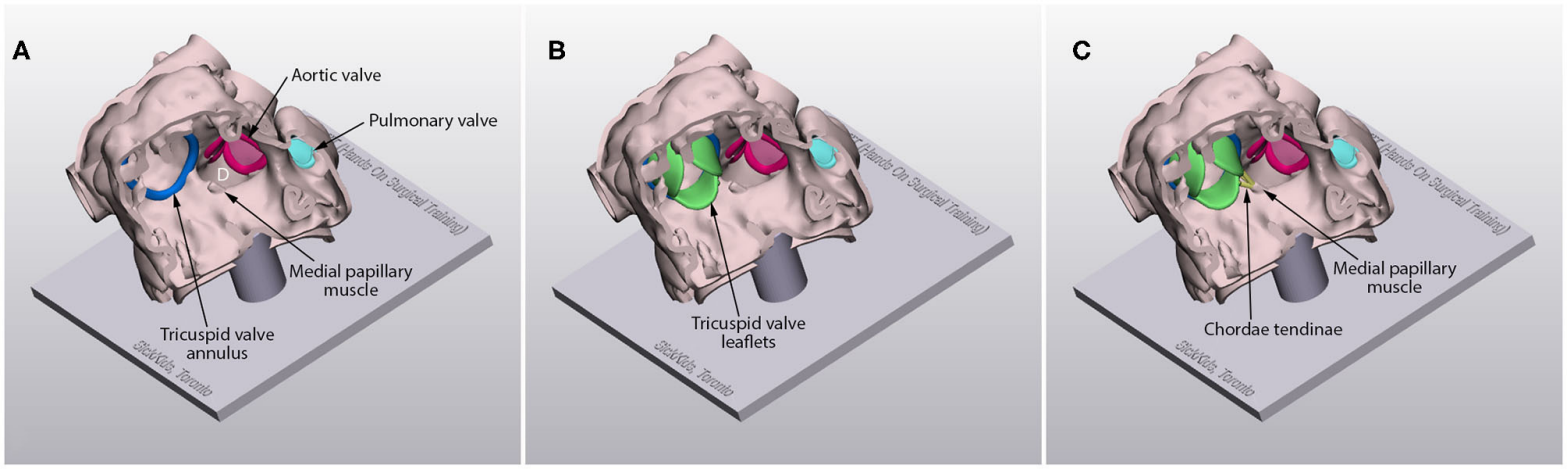
Models for patch repair of a ventricular sepal defect (D) with the hole well-exposed **(A)**, with the graphically designed cardiac valve leaflets added **(B)** and with the graphically designed cardiac valve leaflets and chordae tendinae added **(C)**.

**Figure 4:**
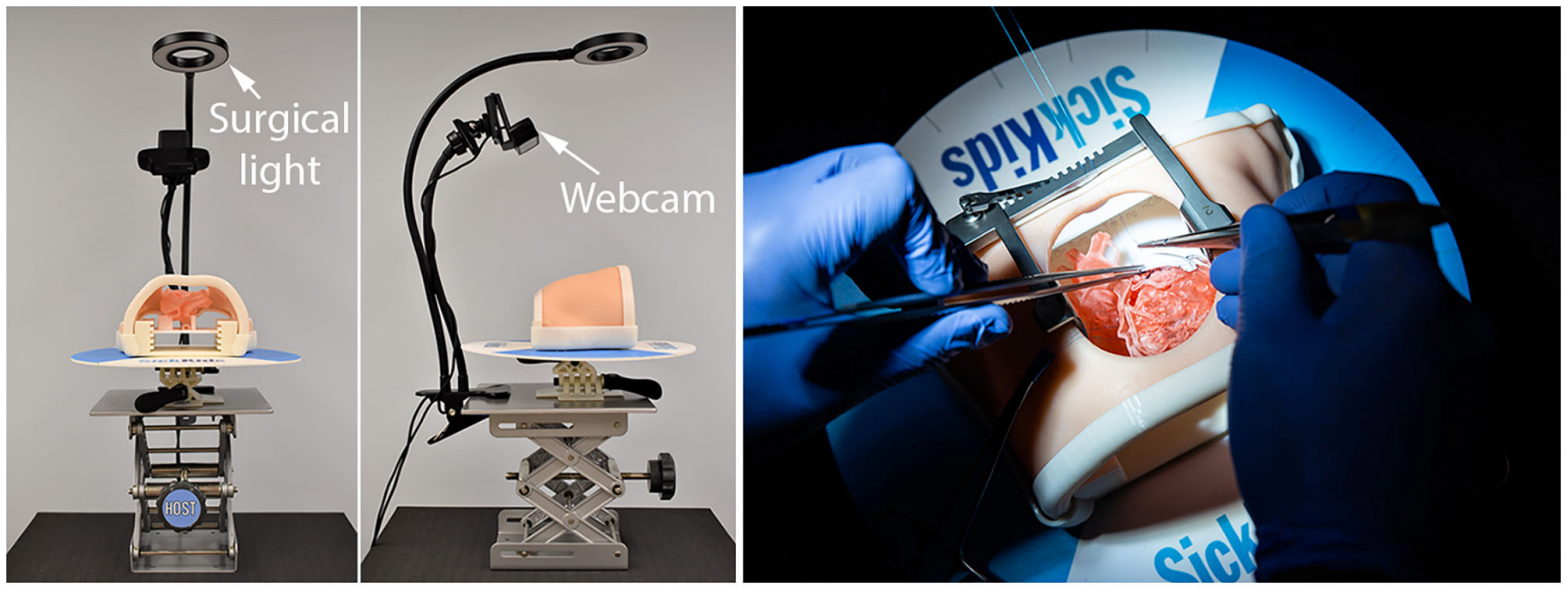
Operating table-chest wall simulator assembly. The simulator is equipped with suture retention disk, surgical lighting and webcam video-recorder. The table can be raised and tilted.

3D-printed models are applicable for training of both extracardiac and intracardiac procedures. They can also be useful in training of minimally invasive surgery such as closure of an atrial septal defect through a lateral thoracotomy. Models can be designed in various forms depending on the purpose of the training. Before any procedure is started, an inexperienced surgeon may want to master the skills for cutting, suturing and cannulation. Such entry level skills can be easily practiced on a sheet or tube made of soft material. The surgical procedures can be practiced in a step-by-step fashion from basic bench top models to complex surgical models. For instance, models for patch closure of a VSD can be made in three different forms: a model with a well-exposed hole, a model with cardiac valve leaflets added, and a model with chordae tendinae as well as valve leaflets added (Figure 3). For most intracardiac procedures, the models are made only for the endocardial surface anatomy to reduce the time and cost for reproduction. However, models with full myocardial thickness can be printed using newly introduced super-soft print material (Tissue Matrix, Stratasys, Rehovot, Israel). As currently available imaging modalities do not provide clear definition of the cardiac valve leaflets and chordae tendinae, graphically designed parts are added to the image-based model as described elsewhere (Figure 3) (1, 11). The more sophisticated forms of the model are provided to an advanced group of surgeons. While most congenital heart surgical procedures are reproducible using the techniques shown above, models for cardiac valve surgery and sutureless repair of pulmonary veins using the patient's own pericardium are more difficult to reproduce albeit not impossible. Typically, the models are mounted on a flat platform with supporting pillars so that the platform is simply plastered on the table. A model can be mounted in a chest wall simulator that replicates the environment and ergonomics of the operating table (Figure 4) (48).

The surgeons' operative performance on 3D-printed models can be objectively assessed by using assessment tools such as the Objective Structured Assessment of Technical Skills (OSATS) with Global Rating Scale (GRS) (49, 50) and the Hands-On Surgical Training–Congenital Heart Surgery (HOST-CHS) tool (51–53). The OSATS-GRS is a widely used tool in surgical simulation that is based on Likert scales for evaluation of generic skills and procedure-specific checklists but shows limited reliability and validity at the specialist level (54). The HOST-CHS tool is a procedure-specific checklist for the key procedural steps, the importance of which is graded using a predefined weight. Each step is assessed in a binary manner for “yes (acceptable)” or “no (unacceptable).” The HOST-CHS tool has been proven to provide higher intrarater and interrater reliabilities than conventional OSATS-GRS for arterial switch operation and Norwood operation (51–53). To assess the general aspects of the surgical procedures, the checklist is broken down into holistic categories including procedural fluency, knowledge of technical aspects of the procedure and respect for tissue. Furthermore, the procedural time is an additional separate component to measure as cross-clamp and cardiopulmonary bypass time is an independent predictor of the outcome in congenital heart surgery especially in young children (55–57). Assessment of operative performance requires video-recording of the procedure. While extracardiac repairs such as arterial switch operation, supravalvular aortic stenosis, coarctation repair and Norwood operation can be recorded without difficulty, intracardiac repairs are difficult to record unless a major part of the ventricular wall is removed to expose the surgical scene (Figure 3). Alternatively, an endoscopic camera can be inserted through a hole in the apex of the ventricle. Ideally, the recorded procedures should be evaluated by experienced surgeons, which is often challenging due to a surgeon's busy daily schedule. The HOST-CHS tool enables non-MD raters to assess the surgeons' procedures with accuracy similar to that of experienced MD raters (51).

### Congenital Heart Morphology Teaching

**Figure 5:**
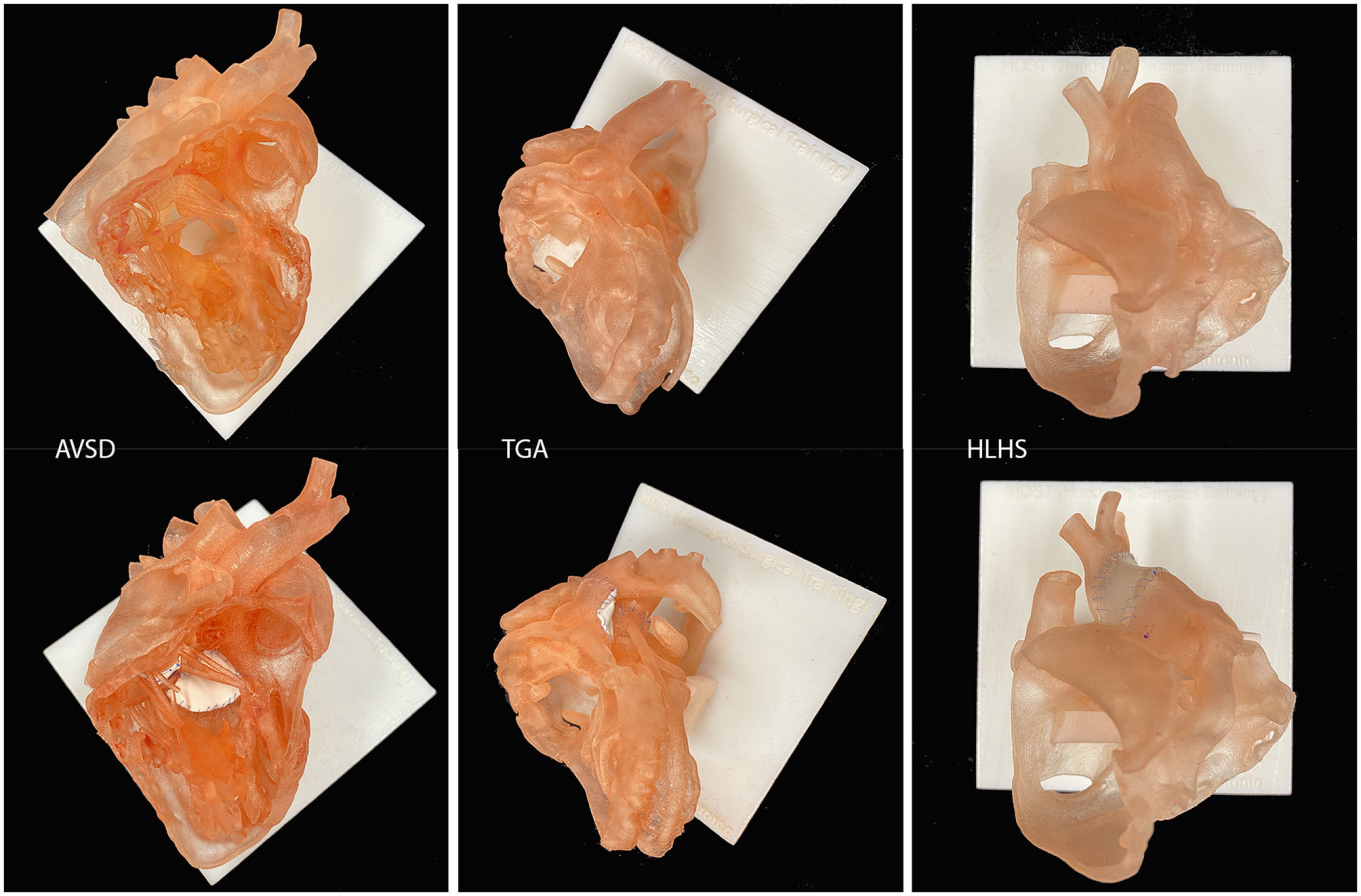
Examples of surgical simulation models for atrioventricular septal defect repair (AVSD), arterial switch operation for transposition of the great arteries (TGA) and Norwood operation for hypoplastic left heart syndrome (HLHS). Only the endocardial surface anatomy is represented with a wall thickness of 0.9–1.2 mm.

Proper understanding of the pathological features of a wide variety of congenital heart diseases is fundamentally important in the accurate diagnosis and surgical treatment of patients. Traditionally, congenital heart morphology teaching has heavily relied on observation of pathologic specimens removed from deceased patients or at cardiac transplantation. However, pathologic specimens are rare resources that are available in the limited number of institutions where specimens have been collected from the old era of high surgical mortalities. While old specimens are deteriorating with time, the sources of new specimens have increasingly been scarce due to ever improving surgical outcomes as well as ethical and legal issues related to retaining human bodies or body parts for educational purposes (58). 3D-printed replicas of congenital heart diseases are extremely valuable educational resources as almost all variations of congenital heart diseases can be reproduced in any number (Figure 5) (59). In contrast to pathologic specimens that can be cut in a limited number of sectional planes, 3D-printed models can be reproduced in any number of cutting planes as well as its entirety for comprehensive demonstration of the complex anatomy. However, representation of thin or small structures such as cardiac valves and chordae tendinae are not satisfactory with currently available imaging and printing technologies. By compensating the weaknesses and limited availability of cardiac specimens, 3D-printed models have emerged as valuable resources for the education of congenital heart morphology (60–66).

### Patient and Family Education

**Figure 6:**
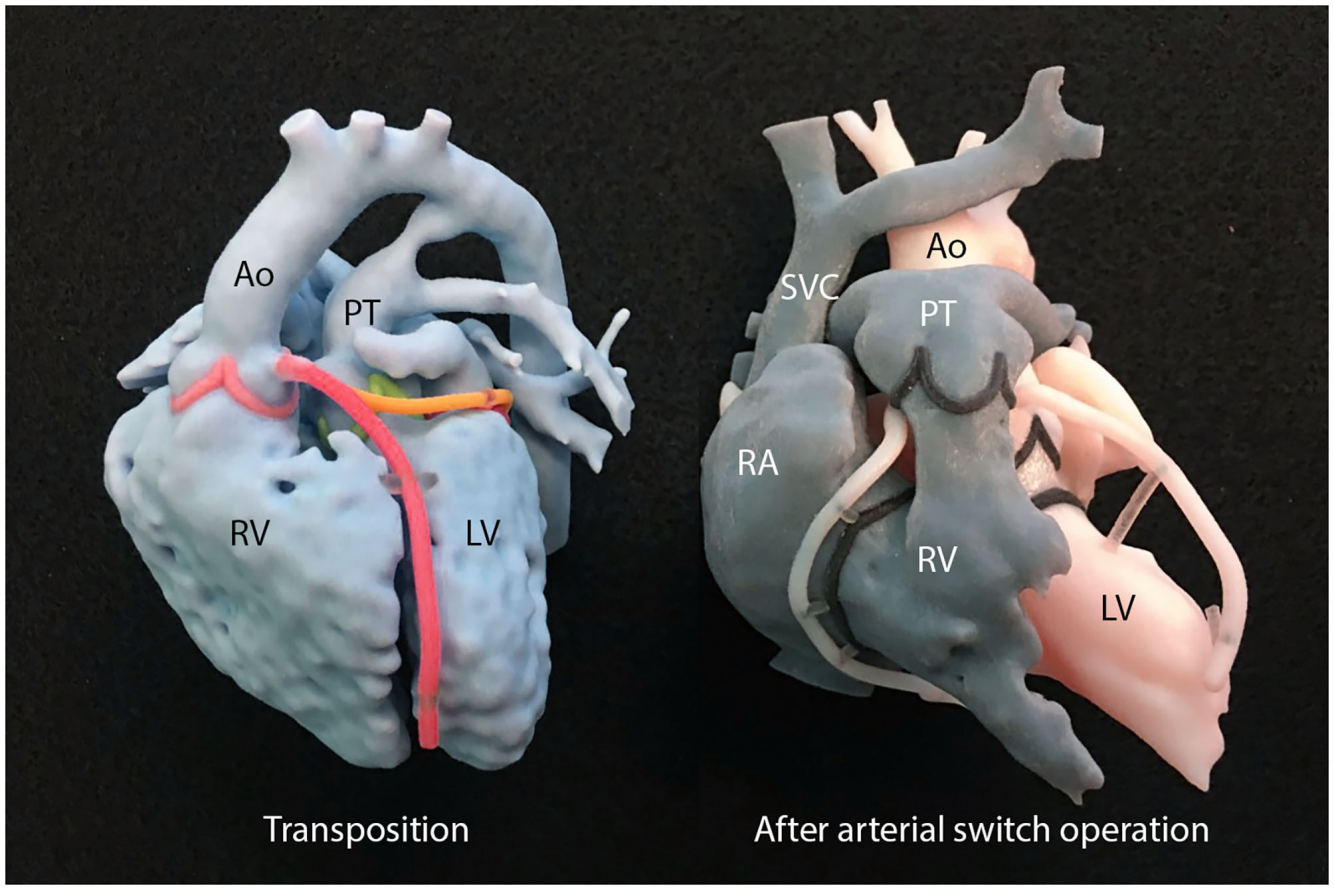
A set of 3D-printed models used for explanation of transposition of the great arteries and arterial switch operation. Ao, aorta; LV, left ventricle; PT, pulmonary trunk; RA, right atrium; RV, right ventricle; SVC, superior vena cava.

3D-printed models of either the patient's own heart or other individuals' with a similar pathology are very helpful for patient counseling (Figure 6) (66). With the physical replicas of the heart on hands, the patients and families can understand the explanation easily with high level of confidence and are readily engaged in the discussion.

## Current Limitations and Future Direction

This information is summarised in Table 2. Despite its utilization for almost two decades, it has been claimed that there has not been enough “scientific” evidence of the usefulness of 3D printing in CHDs (4, 5). Ideally a randomized trial is required. However, the comparison of the surgical outcomes between the groups with and without 3D-printed models is extremely challenging. Such a study would require a large number of patients with CHD due to the rarity and high variability seen in these patients. It is very difficult to scientifically prove the impact of 3D printing in patient management as there are numerous other compounding factors that contribute to patient's outcome. The use of 3D-printed models certainly affects the quality of surgical procedure, cross-clamp time, length of stay in intensive care unit and hospital, required manpower and costs for treatment, each of which would not appear significant individually but may add up to be collectively significant. What matters at this maturing stage of 3D printing is: (1) how to standardize the service, (2) how to maintain quality control of the development and printing process, and (3) how to reduce the costs to an acceptable range.

**Table 2:** Limitations of 3D printing in congenital heart surgery and future direction.

Limitations
• Insufficient objective evidence of the usefulness of 3D printing in congenital heart surgery • Long 3D modeling and printing process • High cost • Limited physical properties of print materials for simulation of surgical procedures • Difficulty in reproduction of cardiac valves and tension apparatuses
Future direction
• Improvement of spatial and temporal resolution of imaging technology • Improvement of printing technology such as high-resolution silicone-based printers and fast printers • Improvement of physical properties of print materials • Insurance coverage of 3D printing as standard medical service • 3D bioprinting

3D printing is an inefficient method of building a 3D object as it requires deposition of thin layers of print material(s), layer upon layer. Using polyjet technology, it takes up to 15 h to print a high-quality model of an adult heart size. The print materials that allow surgical procedures are relatively expensive. Furthermore, post-processing of image data requires the knowledge and hands of experienced specialists as well as expensive software. It is often a painstaking long process. Therefore, a high cost is a major limitation of 3D printing, while it has not yet been recognized as a standard service for reimbursement by the government or insurance companies in most countries. The cost for post-processing software, 3D printing equipment and printing materials will be reduced with increasing utilization of the service as well as technological improvement such as printing time.

Although most surgical procedures are applicable on the models printed with currently available soft materials, further improvements in the materials' physical properties such as elasticity and strength is required. The addition of fiber-like structures to the model may improve the strength as well as the elasticity of the models. Further efforts should be made to simulate the different physical characteristics of cardiac valves, endocardium and pericardium as well as myocardium.

3D printing of cardiac valves and supporting apparatuses is an important area to explore as valve abnormalities are not only common but also surgically challenging. Although accurate replication of the pathology will remain difficult because of the limitation in 3D imaging of the valve leaflets and chords that are constantly moving, delicate structures (67–70), the pathology can be graphically simulated with computer aided design tools based on the combined information from echocardiography and CT (31). This will allow surgical simulation of cardiac valve repair such as the cone procedure for Ebstein's malformation of the tricuspid valve.

The accuracy of anatomical representation in 3D-printed models will continue to improve with improvement of spatial, contrast and temporal resolutions of 3D imaging as well as printing resolution of 3D printers. The evolution of 3D printing technology will follow a path similar to that of digital paper printing. The printer will become smaller and cheaper, and the resolution will become higher. In the future, 3D printers might become as ubiquitous as digital paper printers are now. Furthermore, 3D printing capability could be an element of sophisticated imaging equipment.

3D bioprinting has been an attractive application of 3D printing as bioprinted tissue may provide the characteristics of human tissue with growth potential (6, 71, 72). If the patient's own cells are used, a risk of rejection is eliminated. While traditional methods are based on the 3D printing of scaffolds that allow incorporation of cells to grow, the modern method exploits direct 3D printing of living cells and biomaterials or bioink such as hydrogels and decellularized extracellular matrix as functional scaffolds. Direct printing enables precise placement of the required number of cells and volume of biomaterials. Potential applications of bioprinting range from fabrication of a simple surgical patch to construction of the whole heart for transplantation. Nonetheless, bioprinting has not yet been successful in real patient management. Bioprinting of patient-specific baffles and patches using the patient's own stem cells, preferably sampled at the time of delivery with fetal diagnosis of the pathology, is practically appealing.

The HOST course for congenital heart surgery using 3D-printed models has not yet been widely disseminated partly because of its relatively short history and also due to rather expensive costs. However, young surgeons' effective surgical skill development is worth the current associate cost of the course. The investment will be recovered indirectly from improved short and long term outcomes of congenital heart surgeries performed by well-trained surgeons. The HOST course will turn out to be affordable, cost-effective program with wide utilization of the service as well as reduced costs. With further improvements in the quality and variety of the models and the development of procedure-specific HOST-CHS assessment tools for all applicable procedures, HOST courses will contribute to the improvement and standardization of congenital heart surgery training and potentially contribute toward surgeons' certification examinations.

## Conclusion

3D printing is useful for surgical decision making and simulation, computer-aided desing of surgical baffles and patches, hands-on surgical training and cardiovascular morphology teaching for medical personnel and patients/family. 3D printing is entering the stage of maturation in its use for congenital heart surgery. It is now time for imagers and surgeons to find how to uniquely utilize 3D printing and how to improve the quality of the products for both better patient outcomes and education of CHD morphology and surgery. Although it is relatively expensive, the cost can be recovered indirectly from improvement of patients' surgical outcome and quality of life. Furthermore, hands-on surgical training with 3D-printed models allows efficient education of surgeons in-training. Implementation of HOST courses in congenital heart surgical training programs is not an option but an absolute must.

## Author Contributions

All authors contributed in 3D modeling and printing of congenital heart diseases for patient management, education and surgical training, and approved the content of the article.

## Conflict of Interest

The authors declare that the research was conducted in the absence of any commercial or financial relationships that could be construed as a potential conflict of interest.
